# Supplementary material for: Circulating HPV DNA as a Biomarker for Pre-Invasive and Early Invasive Cervical Cancer: A Feasibility Study
Source: Cancers (Basel). 2023 May 2;15(9):2590. doi: 10.3390/cancers15092590 (PMC10177194; doi:10.3390/cancers15092590)
Supplement: Supplementary file 1 [file cancers-15-02590-s001.zip › cancers-2260470-supplementary materials.pdf]

Table S1. Cost calculation.

	Cost	Reaction size		Cost per sample
Sequencing costs including Proton Chip v2, 520 flows SE and Proton Hi-Q templating kit	2800	50		56
Ion AmpliSeq Library Kit 2.0	5994	192		31.22
Ion Xpress™ Barcode Adapters 1-96 Kit	7103	3840		1.85
Ion Universal Library Quantitation Kit	800	500		3.20
Ion Ampliseq Custom DNA Panel Primer Pools	3551	3000		1.18
Beckman Coulter™ Agencourt AMPure XP	646	600		1.08
Agilent Bioanalyzer High Sensitivity DNA Kit	344	110		3.13
cfDNA RNAse P Quantification by ddPCR				3
			Total reagent cost per sample	131.87

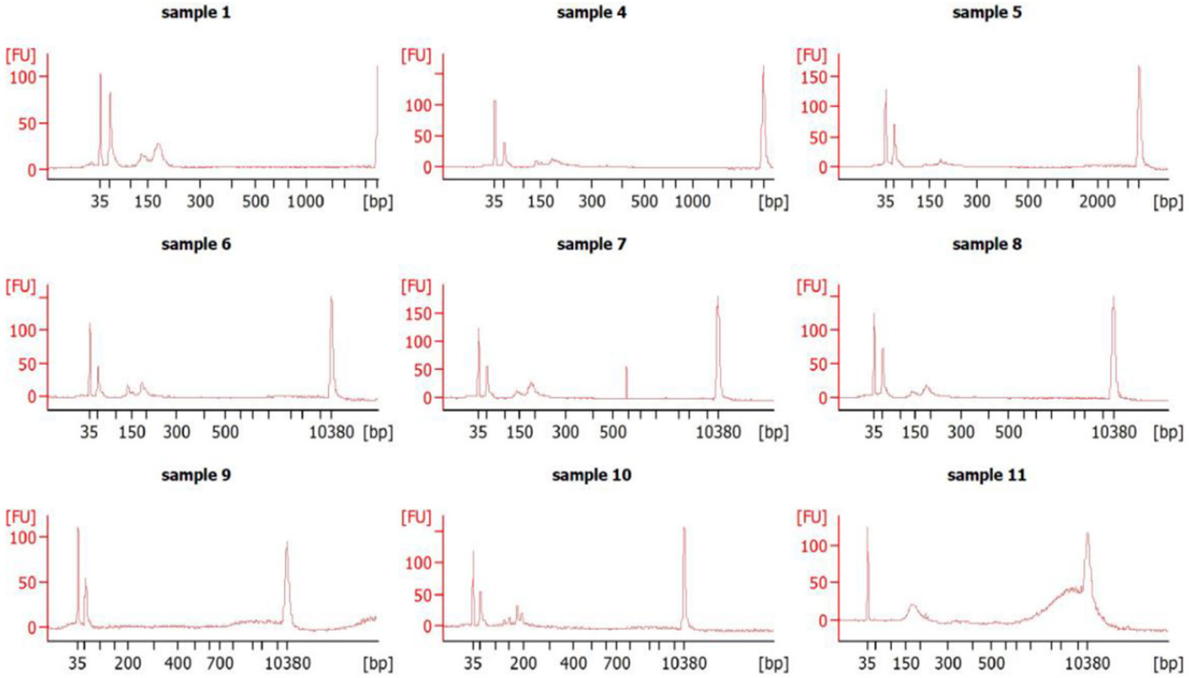

Figure S1. Bioanalyzer traces for 9 libraries

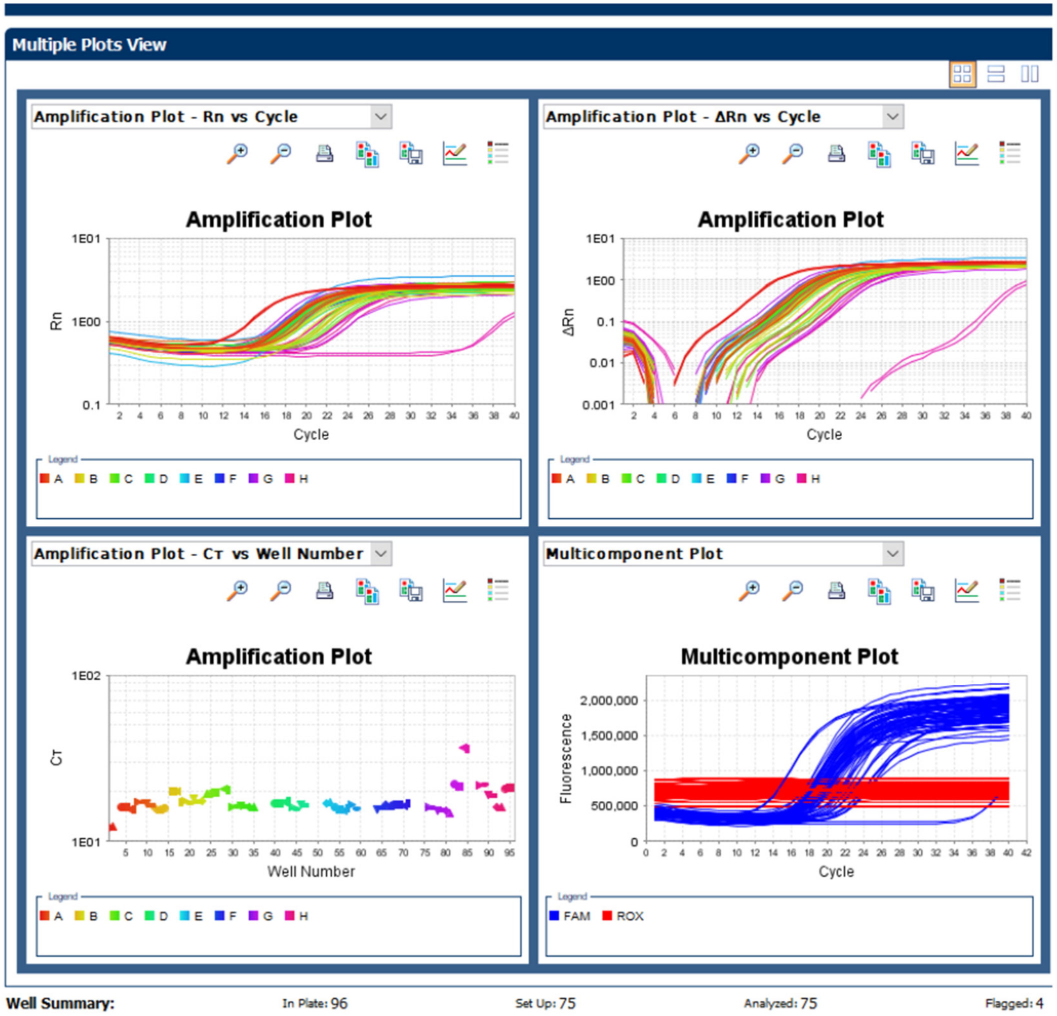

Figure S2. Amplification plots from qPCR of the libraries
